# Calculation of Unknown Preoperative K Readings in Postrefractive Surgery Patients

**DOI:** 10.1155/2018/3120941

**Published:** 2018-02-11

**Authors:** Nicola Rosa, Maddalena De Bernardo, Maria Borrelli

**Affiliations:** ^1^Department of Medicine and Surgery, University of Salerno, Salerno, Italy; ^2^Department of Ophthalmology, Heinrich Heine University Düsseldorf, Düsseldorf, Germany

## Abstract

**Purpose:**

To determine the unknown preoperative K readings (Kpre) to be used in history-based methods, for intraocular lens (IOL) power calculation in patients who have undergone myopic photorefractive keratectomy (PRK).

**Methods:**

A regression formula generated from the left eyes of 174 patients who had undergone PRK for myopia or for myopic astigmatism was compared with other methods in 168 right eyes. The Pearson index and paired *t*-test were utilized for statistical analysis.

**Results:**

The differences between Kpre and those obtained with the other methods were as follows: 0.61 ± 0.94 D (range: −3.94 to 2.05 D, *p* < 0.01) subtracting the effective treatment, 0.01 ± 0.86 D (range: −2.61 to 2.34 D, *p* = 0.82) with Rosa's formula, −0.02 ± 1.31 D (range: −3.43 to 3.68 D, *p* = 0.82) with the current study formula, and −0.43 ± 1.40 D (range: −3.98 to 3.12 D, *p* < 0.01) utilizing a mean K (Km) of 43.5 D.

**Conclusions:**

These formulas may permit the utilization of history-based methods, that is, the double-K method in calculating the IOL power following PRK when Kpre are unknown.

## 1. Introduction

After refractive surgery for myopia, both keratometry and corneal topography tend to overestimate the corneal power, and consequently the calculated IOL power is underestimated, with a high risk of postoperative hyperopia, which may lead to IOL exchange or a piggyback lens [1–9].

This is an important problem because a large number of patients have undergone this surgery with excellent results. Consequently, in the case of a cataract developing, they will expect the same excellent uncorrected visual acuity that they had after refractive surgery, before the cataract onset.

The inaccuracy of the IOL power calculation, excluding an incorrect estimation of axial length [10], is due to several reasons. Among these are pupil width [11], inaccurate measurement of anterior corneal curvature by automated and manual keratometry (K) or computerized videokeratography [12, 13], inaccurate value of the keratometric index resulting from the modified relationship between the anterior and posterior corneal surface, and incorrect estimation of the effective lens position (ELP) resulting from these modifications. To overcome the last problem, Aramberri [14] and Rosa et al. [15] described the so-called double-K method, in which the preoperative K readings (Kpre) are used to estimate the effective lens position. Unfortunately, in most cases, this value is unknown.

The purpose of our study was to find a new method that permits the calculation of the Kpre, when they are not available, and to compare and discuss the results of those obtained by other methods described in the literature [13–16].

## 2. Materials and Methods

This retrospective study was comprised of consecutive patients who had undergone photorefractive keratectomy (PRK) for myopia or for myopic astigmatism. The institutional review board (IRB) approved the retrospective review of records of analyzed data, which had been collected as part of standard of care. Patients gave their informed consent for the surgery.

Patients were asked to discontinue wearing contact lenses for at least 1 month before the last refractive evaluation, which occurred on the day the patients underwent PRK.

Patients with systemic and ocular diseases that might interfere with the corneal healing process [17–22] or with the refractive outcome, such as diabetes, connective tissue disorders, dry eye, uveitis, corneal and lens opacities, and glaucoma, were excluded from the treatment.

All PRK treatments were performed using topical anesthesia (oxybuprocaine eye drops). The lids were opened with a speculum, and the epithelium was debrided by mechanical brush epithelial removal. All treatments were performed with a 193 nm ESIRIS excimer laser (Schwind, Kleinostheim, Germany). Immediately after surgery, a bandage contact lens was applied to the treated eye under sterile conditions; the bandage was not removed until complete reepithelialization. During this period, operated eyes received diclofenac sodium 0.1% eye drops twice a day for the first 2 days, netilmicin preservative-free eye drops until reepithelialization, and preservative-free artificial tears for 1 month after reepithelialization. All patients received clobetasone eye drops for 1 month in a tapered dose as follows: 1 drop 4 times a day for the first week, 1 drop 3 times a day for the second week, 1 drop twice a day for the third week, and 1 drop once a day for the last week.

Before and 6 months after PRK, all patients had a complete ophthalmic examination, including automatic K readings with an IOLMaster (version 4.08.0002; Zeiss, Jena, Germany). The mean of 3 consecutive good-quality measurements for the keratometry was used.

The 6-month postoperative K readings (Kpost) were related to the difference in K readings (Kpost − Kpre) in an attempt to find a correlation formula that could be utilized by subtracting the calculated differences of the Kpost to obtain the Kpre.

A total of 174 consecutive patients (75 males, 99 females) with a mean age of 32 ± 9 years (range: 18 to 56 years) who had undergone myopic PRK were analyzed.

The left eyes of these patients were utilized to identify a regression formula that correlated the Kpost with the difference in K readings, evaluated with the IOLMaster: This formula was used to calculate the Kpre in the right eyes of the same patients, and the differences between the Kpre obtained with this formula and those obtained preoperatively were compared with the differences obtained from the following methods:

1. The effective treatment calculated at the corneal plane (taking into account the preoperative and postoperative spectacle refraction, converted at the corneal plane with the formula [23] SE corneal plane = SE spectacle plane/[1 − (0.012 × SE spectacle plane)]) was added to the Kpost.
2. In 2004, in an attempt to overcome the problem of the underestimation of the IOL power in patients who had undergone PRK, one of the authors of the current study published the formula *y* = 0.7615*x* − 0.6773, where *x* is the difference in refraction at the corneal plane and *y* is the keratometric difference evaluated with the IOLMaster [13], to be used when the effective treatment was known. In the current paper, the authors utilized this formula to calculate a modified difference in K readings to add to the postoperative ones in order to calculate the preoperative readings.
3. A mean K (Km) of 43.5 [16] was used as standard Kpre, and the differences with the preoperative ones were calculated.

Statistical analysis was performed with a Student paired *t*-test, and *p* < 0.01 was considered to be statistically significant.

## 3. Results

**Figure 1:**
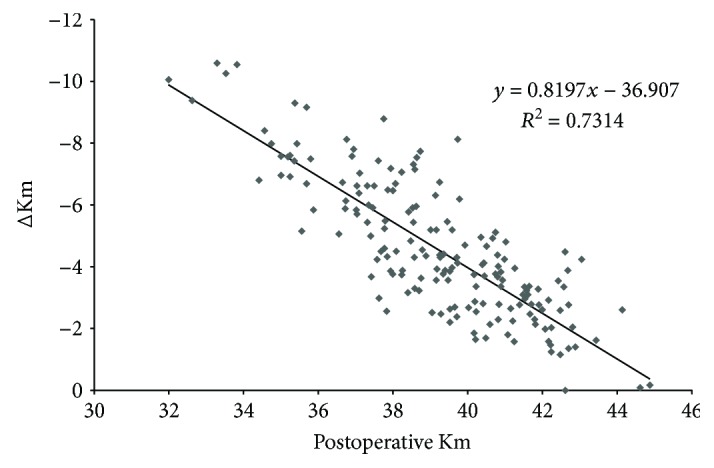
Group B: correlation between the postoperative mean keratometry (Km) and the difference between postoperative and preoperative mean keratometry (ΔKm).

The correlation between the Kpost and the difference between Kpost and Kpre, obtained by analyzing the 174 left eyes with a preoperative refraction of −4.87 ± 2.5 D (range: −10.0 to −0.5 D) and a 6-month postoperative refraction of 0.57 ± 0.81 D (range: −1.88 to +2.5 D), gave the following formula (Figure 1): *y* = 0.8197*x* − 36.907 (where *x* = Kpost and *y* = Kpost − Kpre).

168 right eyes of the same patients (6 of them did not undergo surgery because they were emmetropic) with a preoperative refraction of −5.38 ± 2.86 D (range: −9.33 to −0.5 D) and a 6-month postoperative refraction of 0.49 ± 0.71 D (range: −2 to +1.5 D) were used to test the difference between the preoperative K readings and those calculated with the formula obtained from the left eyes and also with the other above-mentioned methods [5, 13, 16, 23].

Tables 1–4 show that the best results can be obtained mainly with the formula previously described [13] and with the one calculated in the current study, as there is no statistically significant difference with the preoperative K readings (Tables 1 and 3).

In particular, the comparison between these two methods shows that the formula based on the knowledge of preoperative parameters achieves higher percentages of eyes closer to the real preoperative K readings (Tables 2 and 4).

## 4. Discussion

With the third-generation IOL power theoretic formulas (e.g., SRK-T), the AL and K are used to calculate the ELP, which would be automatically underestimated after refractive surgery, as a consequence of K value underestimation [14]. The double-K method described by Aramberri requires a knowledge of the Kpre to estimate the effective lens position in the case of cataract surgery in patients who had previously undergone corneal refractive surgery.

This is an easy task if the Kpre have been recorded, but regrettably, this rarely happens.

Theoretically, if the achieved correction is known, it should be possible to calculate Kpre by adding this value to the postoperative ones. This is not always correct in practice, because it has been shown that the effective treatment does not correspond to the changes detected by the machines; in particular, postoperatively, there is an overestimation of the corneal power, resulting in an underestimation of the effective treatment [24, 25].

Few methods have been described to calculate the preoperative Kpre [13, 16, 26, 27].

In 2004, Rosa et al. [13] described a formula which was not designed with the purpose of calculating the Kpre, but tested in the present paper for such a purpose, and have shown that the results are better than those obtained when the pure effective treatment is used.

In the case that the effective treatment is unknown, we may use a standard mean K [16], or we may use the formula obtained from this study. Although the results of both methods appear to be less accurate than the ones obtained when the effective treatment is known, the results obtained with this new formula are better than the ones obtained utilizing a standard value of 43.5 D.

Recently Saiki et al. [26] described a method to calculate the Kpre, starting from the assumption that it is possible to predict the preoperative anterior corneal power from the postoperative posterior one in patients who had undergone LASIK surgery.

Similar to Saiki, in a recent paper, De Bernardo et al. [27] found no significant changes in the posterior corneal surface after PRK with a good correlation between preoperative anterior Km and postoperative posterior Km. This not only confirms the findings of previously published studies [28–31] but also confirms the possibility of predicting the preoperative anterior Km by utilizing the postoperative posterior one, as stated by Saiki et al. [26].

The results of our present study indicate that it is possible to calculate the Kpre without utilizing the posterior postoperative K readings. Unfortunately, we were not able to compare our data with those of Saiki and De Bernardo because to make such a calculation, the Pentacam is needed, and these data were not available for the patients examined in the present study.

Another limitation of our study is that the formula published in 2004 has been proven to be effective only for the IOLMaster, and at present, we do not know if it can be utilized with other devices. Nevertheless, as the IOLMaster is the most widely utilized device, we believe that this formula can be easily applied.

## 5. Conclusions

In conclusion, we suggest that to calculate the Kpre, if the effective treatment is known, the formula obtained from the study published in 2004 [13] is the best option. If, on the other hand, the effective treatment is not available, both standard mean K and the formula reported in this study may be utilized.

## Figures and Tables

**Table 1:** Differences (in diopters) in 168 right eyes between preoperative keratometry readings and the calculated ones with the knowledge of the effective treatment.

	Effective treatment calculated at the corneal plane	Rosa et al. 2004
Mean	−0.61	0.01
SD	0.94	0.86
Min	−3.94	−2.61
Max	2.05	2.34
*p*	<0.01	0.82

**Table 2:** Comparison between the two methods to calculate the preoperative keratometry readings with the knowledge of the effective treatment.

Range	Effective treatment calculated at the corneal plane		Rosa et al. 2004	
	*n*	%	*n*	%
±0.5 D	65	38.7	94	56
±1 D	146	86.9	150	89.3
±2 D	160	95.2	165	98.2
>±2 D	8	4.8	3	1.8

*n* = number of eyes; % = percentage of eyes; D = diopters.

**Table 3:** Differences (in diopters) in 168 right eyes between preoperative keratometry readings and the calculated ones without the knowledge of preoperative parameters.

	Current study	Preoperative mean keratometry = 43.5
Mean	−0.02	−0.43
SD	1.31	1.40
Min	−3.43	−3.98
Max	3.68	3.12
*p*	0.82	<0.01

**Table 4:** Comparison between the two methods to calculate the preoperative keratometry readings without the knowledge of preoperative parameters.

Range	Current study		Preoperative mean keratometry = 43.5	
	*n*	%	*n*	%
±0.5 D	54	32.1	39	23.2
±1 D	123	73.2	120	71.4
±2 D	160	95.2	156	92.9
>±2 D	8	4.8	12	7.1

*n* = number of eyes; % = percentage of eyes; D = diopters.
